# A high-protein diet for reducing body fat: mechanisms and possible caveats

**DOI:** 10.1186/1743-7075-11-53

**Published:** 2014-11-19

**Authors:** Dominik H Pesta, Varman T Samuel

**Affiliations:** Department of Internal Medicine, Yale University School of Medicine, New Haven, Connecticut USA; Departments of Veterans Affairs Medical Center, West Haven, Connecticut USA; Department of Sport Science, Medical Section, University of Innsbruck, Innsbruck, Austria; Department of Visceral, Transplant, and Thoracic Surgery, D. Swarovski Research Laboratory, Medical University of Innsbruck, Innsbruck, Austria

**Keywords:** High-protein diet, Weight loss, Satiety, Energy expenditure, Thermic effect of food

## Abstract

High protein diets are increasingly popularized in lay media as a promising strategy for weight loss by providing the twin benefits of improving satiety and decreasing fat mass. Some of the potential mechanisms that account for weight loss associated with high-protein diets involve increased secretion of satiety hormones (GIP, GLP-1), reduced orexigenic hormone secretion (ghrelin), the increased thermic effect of food and protein-induced alterations in gluconeogenesis to improve glucose homeostasis. There are, however, also possible caveats that have to be considered when choosing to consume a high-protein diet. A high intake of branched-chain amino acids in combination with a western diet might exacerbate the development of metabolic disease. A diet high in protein can also pose a significant acid load to the kidneys. Finally, when energy demand is low, excess protein can be converted to glucose (via gluconeogenesis) or ketone bodies and contribute to a positive energy balance, which is undesirable if weight loss is the goal. In this review, we will therefore explore the mechanisms whereby a high-protein diet may exert beneficial effects on whole body metabolism while we also want to present possible caveats associated with the consumption of a high-protein diet.

## Introduction

Diets high in protein have been shown to be a potential tool for weight loss [1]. General dietary guidelines for adults suggest an acceptable macronutrient distribution range (AMDR) of 45-65% of total energy from carbohydrates (CHO), 20-35% from fat (F), and 10-35% from protein (P) with a recommended dietary allowance (RDA) of 46 and 56 g/d or 0.8 g/kg body weight (BW) of P for females and males, respectively [2]. A diet is therefore considered high in protein if it exceeds 0.8 g/kg BW or the habitual 15-16% of total energy [3]. High-protein (and low CHO) diets have recently received much attention in form of the Atkins diet which is a non-energy-restricting, low CHO (as low as 30 g/day), high-protein/high-fat diet [4], the South Beach diet (low CHO/high protein diet), the Stillman diet (low CHO/high protein/low fat) or the Zone diet (low CHO/high protein) (Table 1) [5]. But also diets high in protein but containing a normal amount of CHO (20% P, 50% CHO and 30% F) have been successfully used to improve metabolic parameters, suggesting that weight-maintenance depends on the protein content but not necessarily on a low CHO content [6]. Now, millions of people all around the world follow these popular diets. In this critical review, nutrient-specific mechanisms of protein-induced satiety for weight loss and preservation of fat-free mass as well as possible caveats of a high-protein diet will be discussed.

**Table 1:** **Popular high-protein diets and their macronutrient composition**

DIET	CHO	Fat	Protein	g/kg/d Protein*
USDA recommend	45-65%	20-35%	10-35%	0.8
Atkins [4]	6%	59%	35%	2.3
South Beach [5]	28%	33%	39%	2.6
Stillman [5]	3%	33%	64%	4.3
Zone [5]	36%	29%	34%	2.3
High Protein, normal CHO [6]	50%	30%	20%	1.3

*based on a 2000 kcal diet and a 75 kg person.

### Mechanisms of satiation with a short-term high-protein diet

Sustained satiety is a key component to induce a negative energy balance and to promote weight loss. An ideal weight loss strategy would promote satiety and maintain basal metabolic rates despite a negative energy balance and reduction in fat-free mass. Satiety is multifactorial and influenced by many components including but not limited to the endocrine system, the cognitive and neural system as well as the gastrointestinal system. A diet high in protein seems to be able to influence certain systems. The hierarchy for macronutrient-induced satiating efficiency is similar to that observed for diet-induced thermogenesis (DIT): protein is the most satiating macronutrient followed by CHOs and fat, which is least satiating [7]. This satiating effect is most significant after high-protein diets [8]. Based on a visual analogue scale (VAS), which comprises a standard tool to measure subjective appetite and satiety [9], satiety was significantly greater after a 60% protein meal than after a 19% protein meal [10]. These findings were confirmed by Crovetti et al. who reported significantly greater satiety after consumption of an isocaloric meal containing 68% protein compared with a 10% protein meal [11]. Increased satiety helps to decrease energy intake, which is a requisite for successful weight loss. In general, increased satiety has been observed after meals with a protein content in the range of 25% to 81% [12].

Interestingly, different types of protein induce distinct effects on satiety and responses of (an)orexigenic hormones. Also the mechanism of satiety is different, especially for whey and casein proteins. Hall et al. compared whey and casein protein and reported decreased energy intake from a buffet meal offered after 90 min after whey protein ingestion, indicating an increased satiety response to whey [13]. As the absorption of casein is slower due to clot formation, 90 min may be too short to make the conclusion of increased satiety. Veldhorst et al. [14] compared the satiating effects of either 10% or 25% of energy from casein-, soy-, or whey-protein. At 10%, whey provoked the strongest reduction in hunger followed by casein and soy. Decreased hunger rates coincided with higher leucine, lysine, tryptophan, isoleucine, and threonine responses with this protein type. For the 25% protein intake, the authors report larger responses in hormone concentrations for whey-protein as compared to casein- or soy-protein. When Hochstenbach-Waelen et al. [15] compared the effects of 2 diets with either 25% or 10% of energy from casein, they report increased energy expenditure, protein balance, satiety, and negative fat balance with the 25% casein diet as compared to the 10% diet, indicating a dose-dependent satiating effect for this type of protein. Diepvens et al. [16] examined the effects of whey protein, pea protein hydrolysate, a combination of whey protein and pea protein hydrolysate and control milk protein (80% casein and 20% whey) on appetite ratings and satiety hormones. They found that pea protein hydrolysate was most effective in suppressing hunger, followed by whey protein, as compared to milk protein. Surprisingly, these findings do not correlate with changes in satiety hormones, which were stimulated more by milk proteins, indicating that peptide hormone response does not always correlate with perceived satiety. Nieuwenhuizen et al. [17] hypothesized that tryptophan (Trp) which serves as a precursor for the anorexigenic neurotransmitter serotonin, would contribute to the satiating effect of this amino acid in dietary proteins. When comparing alpha-lactalbumin (high in Trp), gelatin (low in Trp) or gelatin with added Trp (high in Trp), the authors report that the breakfast containing alpha-lactalbumin suppressed hunger more than a gelatin or gelatin + trypthophan breakfast. This observation, however, was independent of Trp concentrations. In conclusion, it seems that different types of proteins exert distinct effects on satiety and appetite ratings, mediated by a nutrient-specific secretion of hormones. These differences between the proteins seem to be related to timing and certain amino acid threshold levels.

To assess the nutritional value of dietary proteins considering the protein source and dietary indispensable amino acid content, the digestible indispensable amino acid score (DIAAS) has been established as a new protein quality measure to replace the protein digestibility-corrected amino acids score (PDCAAS) [18]. It is derived from the ratio between the amount (mg) of digestible dietary indispensable amino acid in 1 g of the dietary protein and the amount (mg) of the same dietary indispensable amino acid in 1g of the reference protein. The DIAAS can have values below or in certain circumstances above 100%. Values above 100% should not be truncated as was done for the PDCAAS [19], except when the DIAAS is calculated for protein or amino acid intakes for mixed diets or sole source foods. In general, protein quality, digestibility and utilization by the human body is highest in proteins from animal sources such as meat, milk and egg, followed by legume plant protein such as soy with cereal protein such as wheat concluding this list [20].

Several factors contribute to increased protein-induced satiety in response to a short-term high-protein intake. The most important ones seem to be i) increased energy expenditure, increased concentrations of ii) anorexigenic hormones, and iii) metabolites such as amino acids and iv) altered gluconeogenesis. High protein diets can therefore favorably alter the energy balance equation. Enhanced satiety allows for decreased food intake while an increased thermic effect allows for greater calorie output.

#### Energy expenditure

The thermic effect of food, also called diet-induced thermogenesis (DIT), is a metabolic response to food. Food intake results in a transient increase in energy expenditure attributable to the various steps of nutrient processing (i.e. digestion, absorption, transport, metabolism and storage of nutrients). The DIT is mostly indicated as percentage increase in energy expenditure over the basic metabolic rate (BMR). DIT values are highest for protein (~15-30%), followed by CHOs (~5-10%) and fat (~0-3%) [21, 22]. Based on a recent meta-analysis, the thermic effect of food increases ≈ 29 kJ/4184 kJ of ingested food for each increase of 10 percentage points in the percentage of energy from protein [23]. In other words, if a subject therefore consumes an 8368 kJ/d diet with 30% energy from protein, then the thermic effect of food will be 58 kJ/d higher than if protein contributes only 20% of the dietary energy. This is only an estimate while actual measurements can be higher, as shown below.

The high DIT of protein therefore affects energy balance. Whitehead et al. [24] used a room calorimeter to assess 24-h energy expenditure in subjects on a high-protein diet (36% energy from protein) against two with 15% energy from protein, one high in carbohydrate and the other high in fat. The authors reported that energy expenditure was 297 kJ/d higher in subjects consuming the high-protein diet (P < 0.05), which was in agreement with the increase in sleeping metabolic rate. Mikkelsen et al. [25] found that subjects consuming a diet containing 29% of protein had a 891 kJ/d higher resting metabolic rate than subjects consuming the same eucaloric diet with 11% energy from protein. For weight loss, however, DIT-related satiety is even more important. A high protein diet is associated with increased 24-h diet-induced energy expenditure [26]. The increase in DIT may increase satiety. A potential mechanism to account for this observation is the increase in oxygen demand to metabolize proteins, which can enhance satiety [26]. A similar phenomenon of appetite suppression can be observed at high altitude where oxygen is limited [27]. This increased oxygen demand stems from the high postprandial amino acid oxidation rate which is of even greater importance if amino acids are given in excess of their deposition.

#### Satiety hormones

There are other possible mechanisms to explain the improvement in satiety with high-protein diets. Early observations that glucose was more expediently metabolized after an oral load versus a matched intravenous load led to the discovery and characterization of the incretin hormones. Two key incretins are glucose-dependent insulinotropic polypeptide (GIP) and glucagon-like peptide-1 (GLP-1). These hormones are synthesized in the gut and secreted from enteroendocrine cells in the intestinal epithelium in response to an oral nutrient load [28]. The release of the two incretin hormones potentiates glucose-stimulated insulin release from the beta-cells [29]. Incretins potentiate glucose disposal by stimulating insulin secretion, estimated to account for at least 50% of the total postprandial insulin release [30, 31]. Besides their insulinotropic action, GLP-1 inhibits glucagon secretion in a glucose-dependent manner, thus diminishing postprandial glucose excursions [29]. Extrapancreatic effects of GLP-1 include inhibition of gastrointestinal motility and secretion and thereby regulation of appetite and food intake.

Cholecystokinin (CCK) is a peptide hormone found in the brain and the gastrointestinal tract [32]. CCK stimulates intestinal motor activity and significantly contributes to the inhibition of gastric emptying. Ingestion of dietary protein and especially digestion (hydrolisation) of proteins into amino acids effectively stimulates CCK release in the gut [33]. It is now clear that CCK reduces food intake and meal size and induces satiety [34] in a variety of mammalian species including rats, rhesus monkeys and humans. Brennan et al. [35] reported suppressed energy intake in lean and obese subjects after high-protein meals, an effect potentially mediated by CCK and ghrelin. The short-term meal-related satiety signal of the peptide is most likely mediated through the CCKA receptor [36] and may involve other satiety players such as insulin and leptin [37]. CCKs functions include stimulation of pancreatic secretion, gallbladder contraction and intestinal mobility as well as inhibition of gastric emptying [38].

A recent study showed that GLP-1 blunted postprandial glucose response and reduced insulin release by reducing gastric emptying at physiological doses in response to a mixed meal. This increased gastric retention, lowered hunger and the desire to eat and augmented satiety [39]. The process of gastric emptying might play an important role in the perception of hunger and satiety. In the same study, GIP reduced postprandial glucose increment primarily through an increased insulin release with no effect on the gastric emptying rate [39]. GIP contributes to the regulation of glucose uptake and stimulates triglyceride storage in adipocytes [40]. Chronic exposure to incretin mimetics leads to weight reduction in type 2 diabetics [41]. The ubiquitous serine protease dipeptidyl peptidase-4 (DPP-4) is responsible for the degradation of these peptides. While incretin mimetics do promote weight loss, the effect of DPP-4 inhibitors on weight control is less clear. Nevertheless, pharmacological inhibition of DPP-4 activity may be a strategy to decrease appetite and improve the condition of type-2-diabetes. Inhibition of DPP-4 activity by certain foods is therefore of great importance for medical nutrition therapy. It was shown that the tripeptide Ile-Pro-Ala (IPA) generated by proteinase K mediated proteolysis of the whey protein component beta-lactoglobulin can act as a DPP-4 inhibitor, thereby delaying GIP and GLP-1 degradation [42]. It was suggested by the authors that after luminal degradation, the IPA fragments may act in situ as competitive inhibitors of DPP-4 as it cleaves peptides with an N-terminal alanine or proline amino acid residue [43], suggesting a similar, albeit more modest, effect as pharmacological DPP-4 inhibitors such as Sitagliptin. Increased incretin levels mediate postprandial insulin release, thereby inducing satiety [44] and the preference for food-related cues [45].

The secretion of gut neuropeptides that induce satiation, GLP-1, CCK, and peptide YY (PYY) seem to be increased in response to a high-protein diet whereas concentrations of orexigenic hormones such as ghrelin seem to be reduced [46, 47]. Enteroendocrine cells which release GLP-1 and GIP are in direct contact with the gut lumen and by this means seem to be able to sense arrival and passage of nutrients along the gastrointestinal tract. Studies involving human enteroendocrine cell lines have shown that essential amino acids (after hydrolytic digestion of complex proteins) activated p38 MAPK and ERK1/2 pathway resulting in nutrient-stimulated GLP-1 release [48]. It has to be noted, however, that GLP-1 secretion is nutrient related (increased after a protein meal in combination with CHOs) [49]. These gut neuropeptides were increased in women fed a high protein diet (30% P/40% CHO/30% F) when compared to an adequate protein diet (10% P/60% CHO/30% F) [46]. Increased GLP-1 concentrations were also found in men after a high protein breakfast, lunch and dinner [50]. A study in humans by Blom et al. showed increased satiety due to decreased postprandial ghrelin concentrations in response to a high-protein breakfast compared to an isocaloric high-carbohydrate breakfast [51]. It is very likely that increased protein-induced satiety leads to reduced subsequent energy intake which is beneficial for weight loss. This effect is not related to a conditioned taste aversion. A decreased gastric emptying rate following a high-protein diet has also been observed [52].

#### Plasma amino acid levels

High-protein diets may directly promote a satiety response. In 1956 the aminostatic hypothesis was introduced: increased serum amino acid concentrations produced feelings of satiety whereas decreasing concentrations created feelings of hunger [53]. Diets high in protein will elevate concentrations of plasma amino acids [54]. According to Nefti et al. high intake of protein induces a vagal feedback to the satiety center of the nucleus tractus solitarius in the brainstem and the hypothalamus to suppress hunger [55]. Poppitt et al. [56] showed that the satiating effect after a high-protein preload was significantly larger than preloads containing an iso-energetic amount of carbohydrates or fats. Along these lines, Westerterp-Plantenga [26] found a significant increase in 24-h satiety in subjects consuming a high-protein diet compared to a high-fat diet. The aminostatic hypothesis has been supported by several, but not all studies [57], showing that high-protein diets result in higher levels of satiety, however, complex homeostatic mechanisms between the peripheral organs and the central nervous system which cause the aminostatic effect are not yet fully understood. More research in this area is necessary to elucidate this hypothesis.

#### Gluconeogenesis

Alteration of gluconeogenesis has been found to contribute to satiety [58]. High-protein and low-carbohydrate diets promote hepatic gluconeogenesis to maintain plasma glucose levels. Two key enzymes of gluconeogenesis, phosphoenolpyruvate carboxykinase (PEPCK) and glucose-6-phosphatase (G6P), are upregulated in rats fed a high-protein diet, suggesting that gluconeogenesis is stimulated by a high-protein diet [59]. A modulation of hepatic gluconeogenesis and increased glucose homeostasis could be responsible for the satiating effect in this animal model [60]. Veldhorst et al. [61] also report an increase in gluconeogenesis in healthy men in response to a high-protein diet as compared to a normal protein diet. A recent study in humans found an increased gluconeogenesis following high-protein intake but this increase was unrelated to appetite suppression [62]. Instead, the authors observed an increased production of ketone bodies (especially beta-hydroxybutyrate) in response to the high-protein diet. The increased concentration of beta-hydroxybutyrate may act as an appetite suppressing substrate [62]. The latter may be most important in contributing to increased satiety, especially if the diet is high in protein and low in CHOs. It is also well established that a decreasing level of blood glucose is an appetite stimulating state whereas amino-acid induced gluconeogenesis acts as appetite suppressant preventing hypoglycemia. Central mechanisms include augmented activation of Pro-opiomelanocortin (POMC) neurons and alpha-melanocyte-stimulating hormone and decreased activation of non-POMC neurons upon acute ingestion of a high-protein diet. Intraduodenal protein can activate vagal afferent fibers and after high-protein ingestion c-Fos expression in neurons of the nucleus of the solitary tract was increased [58].

### The effect of a high-protein diet on body composition and weight loss

High-protein diets can help preserve lean body mass during weight loss. Mettler et al. examined the effect of increased dietary protein intake (~2.3 g/kg BW/d) on lean body mass maintenance during hypoenergetic weight loss in athletes [63]. Performance parameters were not affected in the subjects, most likely due to the short study period. They found that though both groups lost similar amounts of fat mass, lean body mass during the short 2 week study period was preserved in the high-protein group compared to the control group (~1 g/kg BW/d). In this study, the authors balanced energy by changing fat intake and not carbohydrate intake as is usually the case. Glucose intake leads to post-prandial insulin secretion. The inhibitory effect of insulin on lipolysis in adipose tissue leads to the postprandial suppression of fat oxidation [64]. This inverse relationship between dietary carbohydrate intake and fat oxidation may explain why Mettler did not observe differences in fat loss between groups whereas others observed reductions in fat mass in non-athletic, overweight subjects on a high-protein (low-carbohydrate) diet [65, 66]. Table 2 shows examples of foods high in protein relative to their carbohydrate and fat content. A recent meta-analysis of 18 randomized controlled trials found that older adults (50+ years) can preserve lean body mass during weight loss more effectively when they consume a high-protein diet [67].

**Table 2:** **High-protein foods relative to their carbohydrate and fat content**

Food	g Protein per 100 g	g Fat per 100 g	g CHO per 100 g
Wheat gluten	75	1.8	14
Textured vegetable protein	48	0	28
Cottage cheese	11	4.3	3.4
Greek yoghurt	10	0.4	3.6
Egg	13	11	1.1
Lentils	9	0.4	20
Edamame	11	5	10
Split peas	25	1.2	60

With regard to the macronutrient distribution, it appears that there is a difference whether protein is increased at the expense of CHO or fat. Increasing protein at the expense of CHOs leads to increased contribution of amino acids to energy expenditure with a concomitant decrease in lipogenesis due to decreased supply of dietary glucose [68] and likely has a negative impact on exercise performance and training intensity [69]. Carbohydrate supply is critical for strength and endurance performance. Athletes should therefore be aware about limited energy intake and maintenance of training levels. For obese subjects, lowering carbohydrate in favor of protein might be advantageous as dietary CHOs might impair fat oxidation [70] whereas low-CHO, high-protein diets reduce adipose tissue development [71]. Higher daily protein intake at the expense of fat intake could substantially reduce total energy intake, which could possibly translate to a healthier weight status [72].

Long-term effects of high-protein diets depend on the population studied as well as the exact composition of the diet but have generally been shown to include weight reduction and weight loss maintenance as well as beneficial effects on metabolic risk factors such total cholesterol and triacylglycerol. Claessens et al. [73] compared a low-fat, high-carbohydrate diet against a low-fat, high-protein diet. The authors conclude that after 12 weeks of diet intervention, the low-fat, high-protein diet was more effective for weight control. Clifton et al. [74] analyzed data from 215 obese subjects which were either assigned to a 12 week high-protein or standard-protein diet. The authors conclude that subjects in the high-protein group had beneficial effects on total cholesterol and triacylglycerol and achieved greater weight loss and better lipid results. In another study, Clifton et al. [75] determined the efficacy of a high-protein and high-carbohydrate intake on the maintenance of weight loss after 64 weeks of follow-up. The authors found no significant difference between groups regarding weight loss. Protein intake in grams derived from the dietary records, however, was directly related to weight loss [75]. Westerterp-Plantenga et al. [76] studied that effect of a 20% higher protein intake (18% of energy *vs* 15% of energy) for subsequent 3 months of weight maintenance after weight loss. They found that higher protein intake resulted in a 50% lower body weight regain over this time, possibly related to increased satiety and decreased energy efficiency [76]. It has to be mentioned, however, that a hypochaloric diet with a high protein content of 20-30% is only relatively high in protein compared to a eucaloric diet with a normal protein level of 10-15% while the absolute amount of protein often does not differ between the two diets [77].

The mechanisms by which increased long-term dietary protein intake regulate body weight are not well understood but are most likely multifactorial. Depending on the diet, lower triacylglycerol levels and hence fat mass loss with a higher-protein diet as well as increased satiety possibly mediated by increased leptin sensitivity have been discussed [75, 78, 79]. Fluid loss related to reduced carbohydrate intake and overall caloric restriction have also been discussed to mediate weight loss [5].

### Possible caveats of a high-protein diet

Metabolomics studies revealed that high intake of branched-chain amino acids (BCAAs, Valine, Leucine, Isoleucine) and aromatic amino acids (Phenylalanine, Tyrosine) may be associated with the development of metabolic diseases [80]. Importantly, this only occurs in combination with a high-fat diet. BCAA supplementation contributes to accumulation of intermediates propionyl-CoA and succinyl-CoA, which are a catabolic byproduct of BCAA degradation. High catabolic flux of these intermediates interferes with appropriate oxidation of fatty acids, possibly by allosteric inhibition of citrate synthase [81] which slows down the TCA cycle, causing buildup of incompletely oxidized substrates such as acylcarnitines. This accumulation leads to mitochondrial stress, impaired insulin action, and finally to perturbation of glucose homeostasis [80]. This connection might be highly relevant as many overweight people worldwide are effectively on a high-fat diet but might as well do weight-training and supplement with BCAAs. Therefore, in people with a high caloric intake from fat BCAA supplementation might exacerbate the risk of metabolic disease.

Diets high in protein pose a potential acid load to the kidneys, mainly as sulfates and phosphates [82]. It was hypothesized that calcium and hence bone mass was lost in order to buffer this acid load [83]. Although the bone-loss hypothesis has been refuted and there is agreement that high-protein diets are actually favorable to intestinal calcium uptake, bone health and bone mineral density [84], the protein-induced acid-load to the kidneys remains, e.g. as sulfuric acid from the oxidation of methionine and cysteine. This phenomenon is especially prominent in diets such as the Atkins diet which can lead to additional acid buildup from ketone bodies in response to reduced carbohydrates and concomitantly increased fat and protein intake. Frank et al. [85] assessed the effect of short-term increased oral protein ingestion on renal function, hemodynamics, and clinical-chemical variables in healthy young men. They reported significant changes in the glomerular filtration rate, the filtration fraction, albuminuria, serum uric acid, and urinary pH values in the high-protein diet group. The authors conclude that renal hemodynamics and renal excretion is altered in response to a short-term, high-protein diet. Although depended on the source of protein, interventional studies in humans have shown that high-protein diets have the potential to increase the risk of calcium stone-formation in the urinary tract [82, 86]. In order to maintain an acid–base balance in the body, people on a high-protein diet should consider ingestion of alkali buffers such as fruits and vegetables high in potassium (alkaline-forming foods). Glutamine or sodium bicarbonate supplements can also help to restore acid–base balance in the body. In general, people experimenting with high-protein diets are advised to monitor their renal function.

People on high-protein diets are advised to choose their source of protein very carefully (i.e. emphasize the use of high-quality protein sources from plant origin). Many protein-rich foods of animal origin (e.g. red meats, eggs and dairy products) also contain high levels of saturated fats and cholesterol. This may put consumers of high-protein diets at higher risk for heart disease, hyperlipidemia and hypercholesterolemia [87]. Healthier proteins from vegetables (soy protein, beans, tofu, seitan or nuts) or fish could be a valuable alternative. Finally, all excess protein will eventually be converted to glucose (via gluconeogenesis) or ketone bodies [88, 89]. This may also explain the increased gluconeogenesis in response to a high-protein diet, as described above. In a state of low energy demand, these metabolites will be stored as glycogen and fat, which is undesirable if weight loss is the goal. Along these lines, weight loss can only be achieved by establishing a negative calorie balance, though this may be more tenable on a high-protein diet.

## Conclusion

Whereas diets high in protein have considerable beneficial effects on satiety and weight control, which is of great interest to e.g. obese individuals, there are some caveats to high protein diets such as increased acid load to the kidneys or high fat content of animal proteins. Awareness of these caveats enables individuals choosing to consume a high-protein diet to get the most benefit from it.
